# Effect of 3-Dimensional, Virtual Reality Models for Surgical Planning of Robotic Prostatectomy on Trifecta Outcomes: A Randomized Clinical Trial

**DOI:** 10.1097/JU.0000000000002719

**Published:** 2022-07-18

**Authors:** Joseph D. Shirk, Robert Reiter, Eric M. Wallen, Ray Pak, Thomas Ahlering, Ketan K. Badani, James R. Porter

**Affiliations:** 1 Department of Urology, David Geffen School of Medicine at UCLA, Los Angeles, California; 2 Department of Urology, UNC Chapel Hill School of Medicine, Chapel Hill, North Carolina; 3 Department of Urology, Mayo Clinic Florida, Jacksonville, Florida; 4 Department of Urology, University of California-Irvine, Irvine, California; 5 Department of Urology, Icahn School of Medicine at Mount Sinai, New York, New York; 6 Swedish Medical Center, Seattle, Washington

**Keywords:** prostatic neoplasms, virtual reality, robotic surgical procedures

## Abstract

**Purpose:**

Planning complex operations such as robotic-assisted radical prostatectomy requires surgeons to review 2-dimensional magnetic resonance imaging (MRI) cross-sectional images to understand 3-dimensional (3D), patient-specific anatomy. We sought to determine surgical outcomes for robotic-assisted radical prostatectomy when surgeons reviewed 3D, virtual reality (VR) models for operative planning.

**Materials and Methods:**

A multicenter, randomized, single-blind clinical trial was conducted from January 2019 to December 2020. Patients undergoing robotic-assisted laparoscopic radical prostatectomy were prospectively enrolled and randomized to either a control group undergoing usual preoperative planning with prostate biopsy results and MRI only or to an intervention group where MRI and biopsy results were supplemented with a 3D VR model. The primary outcome measure was margin status, and secondary outcomes were oncologic control, sexual function and urinary function.

**Results:**

Ninety-two patients were analyzed, with trends toward lower positive margin rates (33% vs 25%) in the intervention group, no significant difference in functional outcomes and no difference in traditional operative metrics (p >0.05). Detectable postoperative prostate specific antigen was significantly lower in the intervention group (31% vs 9%, p=0.036). In 32% of intervention cases, the surgeons modified their operative plan based on the model. When this subset was compared to the control group, there was a strong trend toward increased bilateral nerve sparing (78% vs 92%), and a significantly lower rate of postoperative detectable prostate specific antigen in the intervention subset (31% vs 0%, p=0.038).

**Conclusions:**

This randomized clinical trial demonstrated patients whose surgical planning involved 3D VR models have better oncologic outcomes while maintaining functional outcomes.

**Abbreviations and Acronyms**
3D: 3-dimensional
mpMRI: multiparametric magnetic resonance imaging
MRI: magnetic resonance imaging
PI-RADS®: Prostate Imaging–Reporting and Data System®
PSA: prostate specific antigen
RALP: robotic-assisted laparoscopic radical prostatectomy
SHIM: Sexual Health Inventory for Men
VR: virtual reality

Robotic-assisted laparoscopic radical prostatectomy (RALP) is an operation with a high cure rate, but is accompanied by the risk of nononcologic, or functional, side effects that may affect patient quality of life post treatment.^1–4^ Balancing these functional side effects with oncologic control is paramount, to the extent that the term “trifecta” is used to describe the confluence of cancer control and preserved sexual function and urinary continence.^5,6^ Traditionally, this has been a difficult proposition, owing to the uncertain nature of the anatomy of the prostate and surrounding structures in relation to prostate tumors. Recently, multiparametric prostate magnetic resonance imaging (mpMRI) has gained traction as a means to delineate the anatomy and identify potential areas of prostate cancer for diagnosis.^7,8^ This imaging is integral to fusion biopsy, in which the mpMRI image is fused over the live ultrasound, allowing the surgeon to target their biopsies to the areas of potential prostate cancer.^9,10^ For surgery, these scans are used somewhat less frequently, as they are generally opaque and difficult to read without extensive training.^11,12^ Successfully performing RALP and achieving strong trifecta outcomes depends largely on the surgeon’s understanding of the patient’s anatomy, including the relationship between the cancerous mass(es), capsule of the prostate, bladder, and the nerves providing sexual and urinary function. As such, the lack of easily digestible and comprehensive imaging remains a marked deficit in preoperative planning for RALP.

Recent work has investigated the impact of 3-dimensional (3D) digital imaging on surgical planning for a variety of operations. 3D imaging has been shown to improve understanding of patient anatomy, influence surgical plans and improve patient outcomes.^13–15^ This is particularly of interest for RALP, where the imaging is relatively new, is at baseline difficult to read and where key anatomical structures are intimately related.

In this context, we identified patients undergoing RALP and performed a multi-institutional, randomized, single-blind clinical trial using 3D virtual reality (VR) models generated from preoperative mpMRI scans. We sought to determine if the use of these patient-specific 3D VR models for operative planning would affect key surgical and trifecta outcomes.

## Materials and Methods

### Participant Eligibility, Institutional Review Board Approval and Reporting

**Figure 1.**
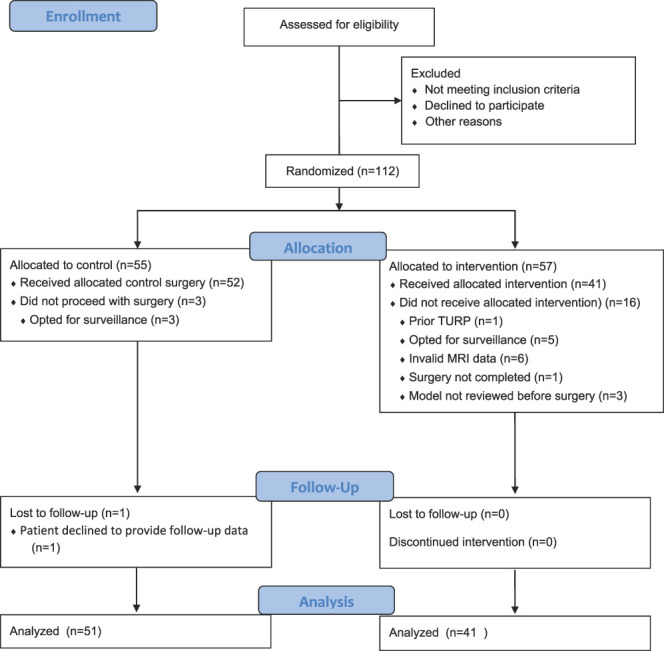
Flow of participants in the 3D VR models for surgical planning of RALP clinical trial. *TURP*, transurethral resection of the prostate.

Participants were recruited from 6 large teaching hospitals. Patients with prior prostate surgery, ablation, radiation or androgen deprivation therapy, or who lacked 3 Tesla preoperative magnetic resonance imaging (MRI) or were unable to give informed consent were excluded from the study. There were no exclusions based on MRI slice thickness. Participants signed a written informed consent document that outlined the specific risks and benefits of enrollment. The study was approved by the Western Institutional Review Board, which served as the Institutional Review Board of record for the study sites (IRB No. WIRB 20171006). The trial was registered on ClinicalTrials.gov (NCT03943368) and followed CONSORT (Consolidated Standards of Reporting Trials) reporting guideline (Fig. 1).

### Randomization, Blinding and Sample Size

Patients were consented and randomized to either a control group, in which the operation was planned with surgeon review of the biopsy results and MRI only, or the intervention group, in which the surgeon reviewed the biopsy results and MRI along with a 3D VR model of the patient’s anatomy that was created by the sponsor from the source MRI. Randomization was stratified by surgeon experience, and each surgeon-specific randomization schedule yielded a randomization in a 1:1 ratio per surgeon. Fifteen surgeons received sequentially numbered opaque sealed envelopes containing the treatment assignment for a single case, which were prepared by the sponsor and provided to each site. Patients were assigned to a study group by opening an envelope at the time of enrollment and were blinded to group assignment.

We calculated the effect size from our previous trials using 3D VR models in robotic partial nephrectomy.^16^ We accounted for within-sample clustering based on surgeon and took 3 values of within-cluster correction (rho=0.3, 0.5, 0.7) to consider low/moderate/high levels of within-cluster correlation, respectively. To account for multiple endpoints and other unpredictable factors, we raised the sample size by 15%. We selected rho=0.5 given the moderate variation in case complexity, which drastically limited within-cluster clustering. Using the above method and paired t-test, the sample size of 90 (45 per arm) provided 80% power to detect a 10% difference in positive margin status with α=0.05.

### Model Preparation, Delivery and Use

**Figure 2.**
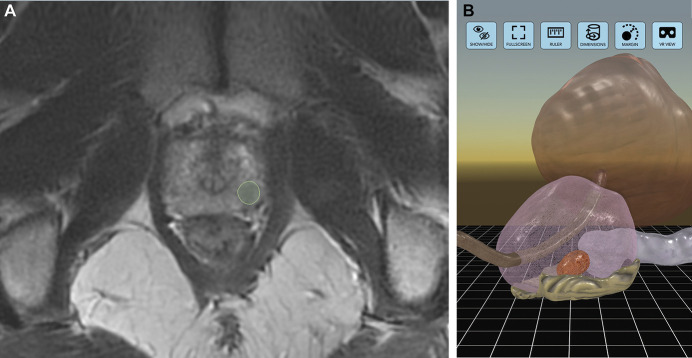
*A*, MRI image of prostate showing lesion (outlined). *B*, 3D model of prostate showing anatomical structures, lesions and color-coded segments from prostate biopsy.

MRI scans from patients randomized to the intervention group were deidentified, provided to the sponsor in DICOM (Digital Imaging and Communications in Medicine) format and used to create a patient-specific 3D VR model for each intervention patient (Ceevra Reveal, versions 2.4–2.8, San Francisco, California). The 3D VR models were generated using this U.S. Food and Drug Administration 510(k)-cleared software, and were comprised of individually segmented and labeled anatomical structures. Masses within the prostate were identified and labeled according to their Prostate Imaging–Reporting and Data System® (PI-RADS®) score. In addition, the biopsy report, which included grade group and location of biopsy, was used to create a patient-specific pathology map that was included in the model to assist the surgeon in understanding the location of biopsy-proven cancer within the prostate. The model included, at a minimum, the prostate, mass(es), bladder, urethra, neurovascular bundles, seminal vesicles, iliac arteries and iliac veins. The prostate was rendered as a wireframe, showing the capsule as a separate structure, and the bladder as semi-transparent to facilitate inspection of the interface between the two and to ensure visualization of the mass(es). Finally, the prostate was divided into labeled segments corresponding to the grade groups of the preoperative biopsy results for those specific segments (Fig. 2).

Prior to their first case, surgeons were trained on the use of the application. Surgeons then reviewed the 3D VR models individually or with their surgical team via a mobile application developed by the sponsor and installed on their smartphones. Parameters such as biopsy results, PI-RADS scores and size for individual lesions were also included within the application. The model could be rotated and zoomed using standard smartphone gestures, and the surgeon could show or hide each anatomical structure during the viewing session. In addition to viewing the models on the smartphone or using a VR headset, the surgeon could view the model picture-in-picture on the robotic console screen using a cable connection between the surgeon’s smartphone and the surgical robot.

### Endpoint Selection and Data Collection

Enrollment and data collection occurred from January 2019 to December 2020. Demographic data were collected for both groups from the medical record, including age and race. Disease parameters collected included preoperative prostate specific antigen (PSA), grade group, clinical stage, lesion size and number, and PI-RADS score, as well as postoperative pathological stage and grade group. Additional preoperative parameters included prostate size, preoperative Sexual Health Inventory for Men (SHIM) scores and the preoperative use of any erectile aids. Clinical data collected included site, surgeon and surgeon experience level, and resident or fellow involvement in the surgery. We also surveyed and recorded when surgeons changed their operative plan after viewing the model prior to surgery. Intraoperative changes in plan unrelated to viewing the model were not recorded to avoid bias.

We observed margin status as defined by the postoperative pathology report nerve sparing (none, bilateral or unilateral) and bladder neck sparing as early operative surrogates for the trifecta of oncologic control, sexual function and urinary function, with nerve sparing and bladder neck as dual surrogates for urinary function.^17^ Partial or tailored nerve sparing was not assessed to maintain heterogeneity among different surgeons. Finally, we observed initial postoperative PSA, SHIM score and urinary pad usage as the gold standard measure of early trifecta outcomes.^6^ These outcomes were observed at 6 months postoperatively. Related to SHIM score, we also recorded the use of any postoperative erectile aid. Initial PSA detectability was chosen as an indicator of poor prognosis, often leading to biochemical recurrence and future additional treatment, and was defined as >0.1, as most sites used standard PSA testing.^18^

### Data Analysis

Data analysis was performed January 2021 through March 2021. As an initial analytical step, we compared baseline characteristics between cases performed with (intervention) or without (control) 3D VR models involved in preoperative planning. We used a 2-sample t-test to compare means between groups and chi-square test or Fisher’s exact test to assess the association between categorical variables.

For the multivariable analyses, we used logistic regression models for the dichotomous outcome measures, where intervention group was the main study variable and control as the referent group. When linear outcomes were dichotomized, we used cut points supported by previous published research.^6^ We independently tested all covariates described above for each outcome measure, resulting in a model controlling for significant variables. Possible interactions between independent variables were tested and models were compared using a likelihood ratio test or by using Akaike information criteria. All statistical tests were 2-sided and carried out at the 5% significance level, using SAS® 9.4 (SAS, Cary, North Carolina).

## Results

From January 2019 to December 2020, 92 patients were enrolled and included in the analysis, with 51 randomized to the control group and 41 randomized to the intervention group. Baseline characteristics were well matched between groups except for a higher number of patients of unknown race in the intervention group (Table 1).

**Table 1.** Baseline characteristics between groups who underwent robotic-assisted radical prostatectomy with and without 3D VR models

Characteristic	Participants		
	Control	Intervention	p Value
No. participants	51	41	
Yrs Age:			
Mean (SD)	62.7 (7.4)	61.7 (7.7)	0.6
Median (IQR)	64 (58–67)	63 (58–67)	
% Race (No.):			
White	78 (40)	66 (27)	0.04
Black	14 (7)	7 (3)	
Other	8 (4)	27 (11)	
Mean preop PSA (SD)	8.6 (6.1)	8.2 (4.7)	0.9
% Preop clinical stage (No.):			
T1c	30 (15)	25 (10)	0.7
T2a/T2bT2c	35 (18)	47 (19)	
T3a	27 (14)	23 (9)	
T3b	8 (4)	5 (2)	
% Preop grade group (No.):			
1-2	59 (29)	75 (31)	0.3
3	8 (4)	5 (2)	
4+	33 (16)	20 (8)	
% Preop risk group (No.):			
Favorable intermediate	39 (20)	37 (15)	0.7
Unfavorable intermediate	16 (8)	22 (9)	
High	45 (23)	41 (17)	
Max PI-RADS score 1–5:			
Mean (SD)	4.2 (0.9)	4.1 (0.7)	0.5
Median (IQR)	4 (4–5)	4 (4-5)	
Prostate cc vol:			
Mean (SD)	40.7 (18)	45.3 (22.4)	0.3
Median (IQR)	38 (28–53)	39 (28–53)	
% Pathological stage (No.):			
T2	53 (27)	63 (26)	0.3
T3a	35 (18)	27 (11)	
T3b	12 (6)	10 (4)	

There were trends toward lower positive margin rates (33% vs 25%) and better urinary continence (1.4 vs 0.9 pads/day) in the intervention group, and no significant difference in nerve sparing, bladder neck sparing and postoperative SHIM score (Table 2). Detectable PSA was significantly lower in the intervention group (31% vs 9%, p=0.036). We also identified the subset of the intervention cases in which the surgeon reported changing the preoperative plan after reviewing the 3D VR model prior to surgery (32% of intervention cases) and recorded the changes reported by the surgeon (Table 3). When this subset was compared to the control group, there was a strong trend toward increased bilateral nerve sparing (78% vs 92%) and a significantly lower rate of postoperative detectable PSA in the intervention subset (31% vs 0%, p=0.038) (Table 4). Using a multivariable logistic model, we found that use of the 3D VR models was a significant predictor of undetectable postoperative PSA (OR=0.23, 95% CI 0.056–0.903, p=0.035), as well as use of the 3D VR models with change in operative plan (OR=0.043, 95% CI 0.002–0.837, p=0.038; Table 5).

**Table 2.** Comparative outcomes between groups who underwent robotic-assisted radical prostatectomy with and without 3D VR models

Outcome	Control	Intervention	p Value
No. participants	51	41	
% Nerve sparing (No.):			
None	6 (3)	12 (5)	0.4
Unilat	16 (8)	10 (4)	
Bilat	78 (40)	78 (32)	
% Margin status (No.):			
Neg	67 (33)	75 (30)	0.4
Pos	33 (16)	25 (10)	
Mean 6-mo SHIM score (SD)	11.7 (7.1)	11.1 (9.5)	0.8
Mean preop SHIM score (SD)	18.6 (5.4)	18.7 (7.4)	0.9
Mean 6-mo pad usage (SD)	1.4 (2.8)	0.97 (1.4)	0.4
% 6-Mo PSA (No.):			
Undetectable	69 (24)	91 (29)	0.036
Detectable	31 (11)	9 (3)	

**Table 3.** Modification in surgical plan after preoperative viewing of 3D VR models

Modification	No. Reported
Unilat to bilat nerve sparing	4
No nerve sparing to unilat nerve sparing	2
Bilat to unilat nerve sparing	2
More aggressive nerve sparing	4
Wider margin at location of mass	3
Altered bladder neck dissection	1
Altered apical dissection	2

**Table 4.** Comparative outcomes between groups who underwent robotic-assisted radical prostatectomy with change in plan after viewing 3D VR models and without 3D VR models

Outcome	Control	Subgroup	p Value
No. participants	51	13	
% Nerve sparing (No.):			
None	6 (3)	0 (0)	0.3
Unilat	16 (8)	8 (1)	
Bilat	78 (40)	92 (12)	
% Margin status (No.):			
Neg	67 (33)	77 (10)	0.5
Pos	33 (16)	23 (3)	
Mean 6-mo SHIM score (SD)	11.7 (7.1)	7.7 (7.7)	0.1
Mean 6-mo pad usage (SD)	1.4 (2.8)	1.1 (1.3)	0.8
% 6-Mo PSA (No.):			
Undetectable	69 (24)	100 (13)	0.043
Detectable	31 (11)	0 (0)	

**Table 5.** Comparative outcomes between groups who underwent robotic-assisted radical prostatectomy with and without 3D VR models, multivariable model

Outcome	Intervention vs Control		Subgroup vs Control	
	Odds Ratio (95% Confidence Interval)	p Value	Odds Ratio (95% Confidence Interval)	p Value
Pos margin status	0.89 (0.293–2.281)	0.7	0.56 (0.109–2.838)	0.5
SHIM score >17	0.92 (0.29–2.913)	0.9	3.93 (0.405–38.045)	0.2
Daily pad usage >1	0.88 (0.299–2.318)	0.7	1.23 (0.255–5.718)	0.7
Detectable PSA	0.23 (0.056–0.903)	0.035	0.043 (0.002–0.837)	0.038

## Discussion

For patients with localized prostate cancer, treatment with RALP presents an interesting challenge.^19,20^ Not only are patients presented with a cancer diagnosis that threatens mortality, but they are also faced with the possibility of sexual and urinary impairment after treatment. To achieve the optimal outcome, all 3 of these factors must be addressed by patient and surgeon.^21,22^ With the advent of mpMRI and the ability of this imaging modality to depict the prostatic anatomy, operative planning has become incrementally easier. However, mpMRI interpretation remains notoriously difficult even for expert radiologists, a barrier which makes it an even less optimal planning tool when used by surgeons.^11,12^ In this context, novel forms of surgical planning such as 3D VR models that precisely depict the size, orientation and proximity of the mass, capsule, nerves and other key structures can help improve the surgeon’s understanding of the patient’s anatomy. In this setting, our study has several important findings.

For patients undergoing RALP, we identified improvements in key postoperative metrics that define oncologic outcomes. Lower positive margin rates and postoperative PSA define better oncologic outcomes, with patients who have either positive margins or early detectable PSA significantly more likely to progress to more advanced disease and require future treatment, such as radiation and/or androgen deprivation therapy.^18,23^ While we did not see a significant difference in margin status, the difference noted in postoperative PSA may be due to varying degrees of margin positivity. As we only defined margins as positive or negative, the degree of positivity may have been less in the intervention cases, and potentially not clinically significant, as a result of the decisions made after review of the 3D model.^24^

While oncologic outcomes in the study improved, it was not at the expense of the functional side of the trifecta—sexual and urinary outcomes. Notably, surgeons were often more aggressive with nerve sparing after viewing the 3D VR models, meaning the surgeon was likely able to precisely select of the right operative pathway regarding margins while optimizing nerve sparing. Simply taking wider surgical margins to improve oncologic outcomes would result in a decreased amount of nerve sparing and bladder neck sparing, and thus decreased erectile function and continence.^17^ Followup values for SHIM score and pad usage revealed there was no functional consequence from better oncologic control.

These findings are likely related to the uncertainty experienced by surgeons reviewing mpMRI and the highly technical nature of these images. More broadly, this represents a deficit of the human ability to reconstruct 2-dimensional images into 3D.^25,26^ Additionally, the 3D VR models likely reduced the cognitive load for surgeons by depicting only the specific information that the surgeon needed to process, while adding the crucial biopsy results to the image. While mpMRIs depict each element of the patient anatomy captured during the imaging process, many of these details are not pertinent to surgical decision making.^27^ The 3D VR models, in contrast, both excluded structures less relevant to the operation and improved understanding of the borders, junctions, shapes and relationships among structures while adding biopsy results.^28^

Our findings will impact care in several ways. First, in the treatment planning stage the surgeon can counsel the patient more directly regarding projected trifecta outcomes. This may lead to treatment selection that is most closely aligned with patient preferences and appropriate patient expectations. Second, the 3D VR models augment the surgeon’s ability to deliver excellent surgical care by addressing key limitations in the current imaging and surgical planning standard of care, allowing this operation to be performed with a more refined balance of oncologic and nononcologic outcomes. Finally, these patients may ultimately avoid further damaging treatment for their prostate cancer, such as adjuvant or salvage radiation and/or androgen deprivation therapy.

Our study has several limitations. First, our positive margin rate is at the upper end of the range reported for this operation.^29^ However, the overall rate is similar to other similar cohorts with high rates of advanced disease.^30^ Second, we noted a trend toward higher postoperative pathological stage in the control group. However, risk groups, a more robust measure of severity, were similar between groups, and the multivariate model individually tested these variables and included them when appropriate. Finally, our study followup was heavily impacted by the COVID-19 pandemic. Postoperative followup with patients, and thus 6-month data collection, was difficult, even as many visits were converted to video visits.

## Conclusions

In patients undergoing RALP, the use of 3D VR models for operative planning improved oncologic outcome while maintaining functional outcomes. Further work may focus on the addition of other anatomical structures and use of the 3D models as a training tool. Additionally, intermediate- and long-term outcomes in these patients should be assessed to ensure the observed effects are durable.
